# First Report on the Occurrence of Tetrodotoxins in Bivalve Mollusks in The Netherlands

**DOI:** 10.3390/toxins10110450

**Published:** 2018-11-01

**Authors:** Arjen Gerssen, Toine H. F. Bovee, Mirjam D. Klijnstra, Marnix Poelman, Liza Portier, Ron L. A. P. Hoogenboom

**Affiliations:** 1 RIKILT, Wageningen University and Research, Akkermaalsbos 2, 6708WB Wageningen, The Netherlands; Toine.Bovee@wur.nl (T.H.F.B.); Mirjam.Klijnstra@wur.nl (M.D.K.); Liza.Portier@wur.nl (L.P.); Ron.Hoogenboom@wur.nl (R.L.A.P.H.); 2 Wageningen Marine Research, Korringaweg 7, 4401NT Yerseke, The Netherlands; Marnix.Poelman@wur.nl

**Keywords:** tetrodotoxins, bivalve mollusks, marine biotoxins, LC-MS/MS, neuroblastoma bioassay

## Abstract

Tetrodotoxin (TTX) is traditionally associated with seafood from tropical regions, but recently TTX was detected in bivalve mollusks in more temperate European waters. In The Netherlands it was therefore decided to monitor TTX in shellfish harvested from Dutch production areas. All shellfish production areas were monitored in 2015, 2016 and 2017. Samples were analyzed using liquid chromatography coupled to tandem mass spectrometry (LC-MS/MS). In total 1063 samples were investigated, and the highest concentrations were observed in 2016, i.e., 253 µg TTX/kg in oysters and 101 µg TTX/kg in mussels. No TTX analogues, with the exception of 4-epi-TTX in one single sample, were found and contaminated samples also showed positive results in the neuro-2a bioassay. The occurrence of TTX seems to be consistent over the last three years with the highest concentrations observed annually in late June. The causative organism and the reasons why specific Dutch production areas are affected while others are not, are still unclear. Initially in The Netherlands an action limit of 20 µg TTX/kg was used to ensure the safety of consumers (2016), but recently The European Food Safety Authority (EFSA) established an acute reference dose, and based on a high portion size of consuming 400 g mussels, this dose was translated into a safe concentration of 44 µg TTX per kg for shellfish. This concentration is now used as an action limit and TTX is formally included in the Dutch shellfish monitoring program.

## 1. Introduction 

Consumption of bivalve mollusks such as oysters, mussels and clams contaminated with marine biotoxins may lead to severe intoxications [1]. Well-known intoxication syndromes are amnesic shellfish poisoning (ASP), diarrheic shellfish poisoning (DSP) and paralytic shellfish poisoning (PSP). Intoxications can also be caused by consumption of fish contaminated with marine biotoxins. Examples are ciguatera fish poisoning and pufferfish poisoning [2,3,4]. Marine biotoxins are produced by specific algal and/or bacterial strains and accumulate in shellfish through filter feeding and in fish through the food web. Toxins responsible for PSP and pufferfish poisoning are saxitoxins (STXs) and tetrodotoxins (TTXs) respectively. Both STXs and TTXs act as blockers of voltage gated sodium channels (VGSC) as present in e.g., excitable neuronal cells [5]. In mild cases intoxications by these two toxin groups will lead to nausea, vomiting, numbing in the lips and mouth. More severe cases lead to paralysis of the motoric muscles leading to respiratory problems. In these cases, patients need to be hospitalized and placed under respiratory assistance [6]. In extreme cases or where respiratory assistance is too late, consumers die [7]. Strict legislation is established in order to avoid such intoxications. For PSP toxins, shellfish production sites are regularly monitored and shellfish areas are closed when concentrations of these toxins exceed the regulatory limit, which in the EU legislation is established at 800 µg STX-2HCl-eq/kg edible shellfish [8]. For puffer fish, EU legislation is clear: fish from the families *Tetraodontidae*, *Molidae*, *Diodontidae*, and *Canthigasteridae* (which are the main species containing TTXs) are not allowed to be placed on the market [8]. Official control for the presence of PSP toxins in shellfish is based either on a mouse bioassay (MBA) or on high performance liquid chromatography with fluorescence detection (HPLC-FLD) [9]. The MBA is currently the EU reference method, but this will change in the coming year [10]. For TTXs, as the expected TTX containing species are forbidden, no official control programs and methods for toxin analysis are established in the EU. Till recently, TTXs were solely associated with specific fish species, snails, and newts [11]. Since 2015, studies have shown that TTXs might end up in shellfish such as oysters and mussels in Europe too.

Within the EU there is a strong tendency to move away from the MBA as the reference method for various classes of marine biotoxins. LC-MS/MS became the reference method for lipophilic marine biotoxins and this will be followed by the possible change towards the HPLC-FLD as the reference method for PSP toxins in shellfish. However, moving from the MBA, which is based on the effect of toxins, towards an HPLC-FLD method, which is based on physiochemical properties, will result in discrepancies. It is well known that the MBA results in false positives, for example due to the presence of high concentrations of zinc in shellfish, specifically oysters [12]. On the other hand, the MBA is probably able to protect consumers for emerging risks, such as recently for TTXs in shellfish in Europe. The first reports of TTXs in shellfish in Europe were from Greece [13]. Unexplained MBA results were further investigated. The initial follow-up method based on the HPLC-FLD for the detection of PSP toxins did not reveal the presence of any STXs. Therefore, a more sophisticated analytical method based on liquid chromatography coupled to tandem mass spectrometry (LC-MS/MS) was applied. This method showed the presence of TTX in shellfish at concentrations ranging between 61 and 195 µg/kg. The presence of TTXs in the Aegean Sea was not a complete surprise, as TTX containing fish were already observed in the Mediterranean sea, most likely migrated through the Suez channel [14]. However, that could not explain the presence of TTX in shellfish. The occurrence of TTX in more temperate waters such as in the United Kingdom was even more surprising, as these toxins were thought only to be present in warmer climates. A survey held in the UK with LC-MS/MS, published early 2015, showed the presence of TTX in shellfish at concentrations as high as 137 µg TTX/kg [15]. A follow-up study showed a maximum concentration of 253 µg TTX/kg in oysters [16]. Moreover, bacterial strains suspected to be responsible for the production of TTXs were successfully identified and positive for TTXs when analyzed [15]. These findings in temperate waters initiated a survey in The Netherlands in 2015 to analyze all shellfish samples harvested for the official control for lipophilic marine toxins also for the presence of TTXs. In this paper, the findings of TTX in Dutch production areas in 2015 as well as the seasonal occurrence in 2016 and 2017 are presented.

## 2. Results

### 2.1. Sanitary Survey Results

**Figure 1:**
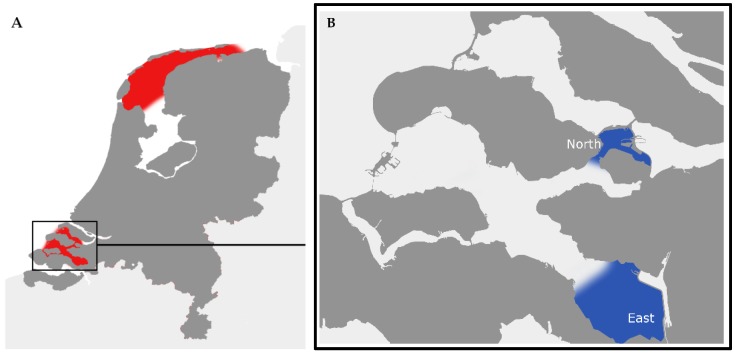
Various shellfish production areas in The Netherlands for mussels and oysters: (**A**) in red an overview of the production locations for mussels and oysters in The Netherlands; (**B**) in blue the areas with a higher prevalence of TTX in the Eastern Scheldt in the South-West of The Netherlands. Based on samples of the official national shellfish food safety program.

After the publication by Turner et al. in 2015, a survey on the occurrence of TTX in shellfish produced and harvested in Dutch production areas was organized. The samples for these TTX analysis were already collected in the framework of the official control of the regulated marine biotoxins (i.e., lipophilic toxins including DSP toxins, azaspiracids, PSP toxins and ASP toxin). None of the samples collected exceeded the regulatory limits for these regulated toxins. In fact, no detectable concentrations of PSP toxins were found and only two shellfish samples were found to contain detectable concentrations of DSP toxins, at respectively 12 and 11 µg okadaic acid (OA)-eq/kg. These concentrations are far below the regulated concentration of 160 µg OA-eq/kg. From all collected samples a sub-portion, stored at −20 °C, was investigated for the presence of TTX. The applied LC-MS/MS method was based on the method published by Boundy et al. [17]. In total 257 samples were analyzed from 14 different production sites (Figure 1A shows the mussel and oyster production sites). Four different species were investigated, 183 mussel (*Mytilus edulis*), 41 oyster (*Castostrea gigas* now *Magallana gigas*), 20 razor clam (*Ensis* sp.) and 13 cockle samples (*Cerastoderma edula*). TTX was detected above the limit of detection (LOD) in 15 samples, 7 mussel and 8 oyster samples harvested in the Eastern Scheldt east and north (Figure 1B), and in 5 of these cases above the limit of quantitation (LOQ) of 20 µg TTX/kg. The highest concentration of TTX was found in an oyster sample collected in the Eastern Scheldt east (124 µg TTX/kg). In the absence of any international health-based guidance values, the findings of TTX in shellfish led to a first risk assessment by the National Institute for public health and the environment (RIVM) and RIKILT Wageningen Research Front office. It was concluded that in the absence of suitable data on toxic concentrations in humans, there should be a zero tolerance for TTX. In practice, this meant that the LOQ of the analytical method, i.e., 20 µg TTX/kg was applied as a temporary decision limit. At the same time, The Netherlands presented the data to the European Commission and asked them to request a risk assessment from European Food Safety Authority (EFSA).

In 2016 all production areas as pointed out in Figure 1A were investigated. In total 403 samples were analyzed in the sanitary survey program, 280 mussel, 60 oyster, 38 razor clam and 25 cockle samples. Sampling was done on a regular basis and was weekly from June till October. When levels were above the LOQ, measures by the official authorities were taken. TTX was found and again solely in the Eastern Scheldt east and north (Figure 1B) and in the same period as in 2015 (June). TTX was detected in 36 samples, 17 mussel and 19 oyster samples, and 20 of these samples contained concentrations above the LOQ of 20 µg TTX/kg. The highest concentration of TTX was found in an oyster sample at 253 µg TTX/kg. TTX concentrations in mussels were much lower, with a maximum of 42 µg TTX/kg. It should be noted that the mussels were not harvested from adjacent plots with oysters but were harvested from the northern part of the Eastern Scheldt while the oysters were harvested from the eastern part of the Eastern Scheldt (Figure 1B). After the first positive finding, the sampling scheme for the Eastern Scheldt was intensified, i.e., more samples were collected in order to follow the TTX concentrations during the toxic episode. The results showed that the concentrations tended to decline rapidly, and the toxic episode lasted for only 4 weeks. For the other regulated toxins, no samples were above the regulated limit. Only a low concentration of 5.3 mg/kg was detected with LC-MS/MS for the ASP toxin domoic acid, i.e., well below the regulatory limit of 20 mg domoic acid/kg.

In 2017 the monitoring was continued in all production areas. Again, a total of 403 samples was analyzed, 281 mussel, 61 oyster, 36 razor clam and 25 cockle samples. The Eastern Scheldt east and north were not affected as much as in 2016. TTX was detected above the LOD of 3 µg TTX/kg in 18 samples, 8 mussel and 10 oyster samples, and in 6 cases (3 mussel and 3 oyster) concentrations were above the LOQ of 20 µg TTX/kg. Again, the highest concentration was found in an oyster sample collected in June (51 µg/kg).

**Figure 2:**
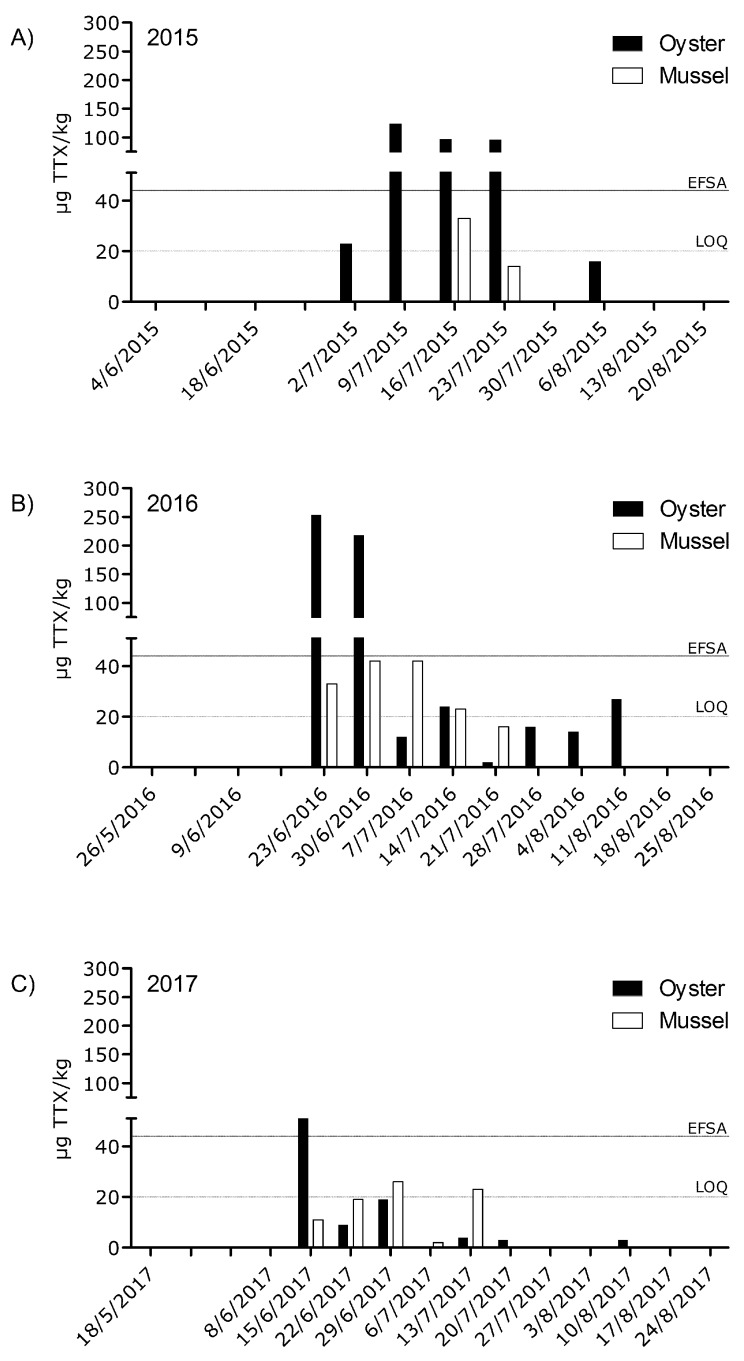
TTX concentrations found in oyster and mussels harvested from the sanitary survey plots in the Eastern Scheldt in respectively (**A**) 2015, (**B**) 2016 and (**C**) 2017. Oysters are harvested in Eastern Scheldt east and mussels were rope cultured mussels in the Eastern Scheldt north. Dates on the x-axis indicate the sampling date of the sanitary survey monitoring program. The EFSA line represents 44 µg TTX-eq/kg and the LOQ 20 µg TTX-eq/kg. Furthermore, the bars displayed below the LOQ (above the LOD) are indicative concentrations more uncertainty will be present in these concentrations.

Figure 2 summarizes the results for the occurrence of TTX in 2015, 2016 and 2017. In the figure also values below the LOQ are showed as indicative values, to get some information about background occurrence of TTX. As already mentioned, only two sampling areas in the Eastern Scheldt were affected, i.e., east and north (Figure 1B). The species sampled in Eastern Scheldt north were rope cultured mussel and in the Eastern Scheldt east oysters. For the rope cultured mussels, samples taken for the official control program were pooled samples with equal numbers of shellfish from top, middle and bottom of the rope. However, in several occasions top, middle and bottom were analyzed individually to determine if there are variations between sampling heights. No significant difference was observed between samples taken from the three different positions. For example, on the 14 July 2016, the sample from the top, middle and bottom contained, respectively, 26, 26 and 20 µg TTX/kg. As from all the data collected it seemed that oysters were more susceptible for TTX than mussels, additional mussel samples were taken from the Eastern Scheldt east as close as possible (based on occurrence) to the oyster production plots. Analysis confirmed that oysters contain indeed higher concentrations of TTX than mussels, as for example on the 29–30th of June 2016 both mussel and oyster samples harvested from adjacent shellfish plots showed concentrations of, respectively, 101 and 218 µg TTX/kg. This difference between mussels and oysters was observed over the course of 2015 to 2017, Figure 2.

### 2.2. Verfication of the Results Obtained

**Figure 3:**
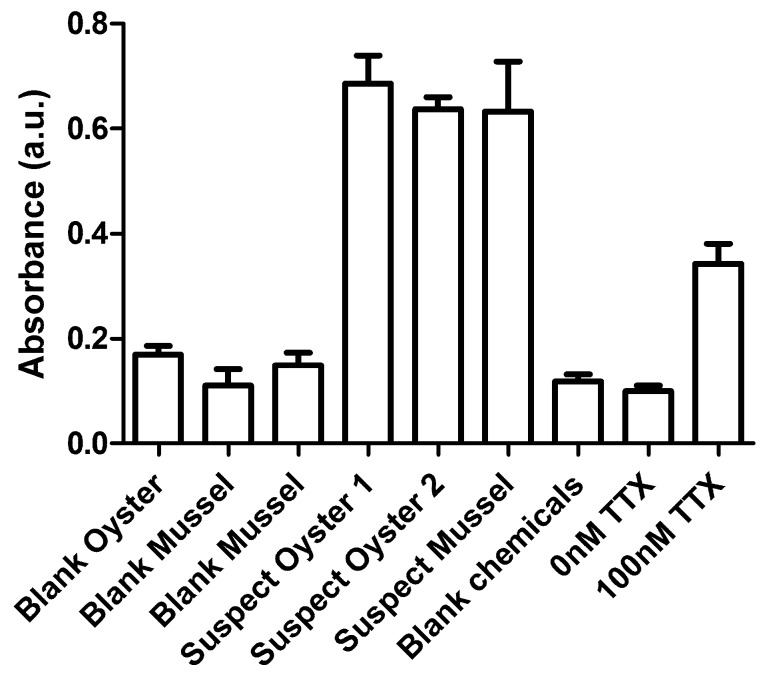
MTT activity of neuroblastoma cells exposed to a pure TTX standard (100 nM), a DMSO control (0 nM), a chemical blank (control) and a subset of contaminated and blank shellfish samples. According to LC-MS/MS analysis, oyster 1 contained 253 µg TTX/kg, oyster 2113 µg TTX/kg and the mussel sample 171 µg TTX/kg. The MTT activity is measured by the absorbance of the purple colored formazan formed by the neuro-2a cells. Results are mean absorbance with the standard deviation (n = 3). Cells are co-exposed to o/v which reduces the MTT activity, which is than counteracted by TTX.

To verify our first positive LC-MS/MS findings and as well to test the applicability of an alternative method for the mouse bioassay, a subset of samples was also analyzed with the neuroblastoma (neuro-2a) bioassay. This assay was shown to be sensitive for this toxin [18,19], but thus far no reports on the testing of fish or shellfish have been published. The principle of this bioassay is based on the cell’s MTT (3-[4,5-dimethylthiazol-2-yl]-2,5 diphenyl tetrazolium bromide) activity assay following exposure to the samples. Neuroblastoma cells were therefore treated simultaneously with sample extracts and with ouabain and veratridine (o/v), the latter leading to an increased sodium influx in cells and resulting in reduced MTT activity. The presence of voltage gated sodium channel blockers such as TTX will reduce the sodium influx as induced by o/v, resulting in more viable cells and thus less reduction of MTT activity. Based on the LC-MS/MS results, two contaminated oyster samples, one contaminated mussel sample and three blank shellfish samples were selected. The contaminated oysters contained 253 and 113 µg TTX/kg and the mussel sample contained 171 µg TTX/kg. The assay was developed as such that a concentration of 20 µg TTX/kg shellfish could be detected. Extracts were prepared, and cells were exposed to these extracts. Results of the neuroblastoma bioassay as shown in Figure 3 clearly indicated that the three contaminated samples are classified as suspect by the bioassay, i.e., showing an increased MTT activity as measured by the absorbance of the formed purple colored formazan. The blank shellfish samples and chemical blank and dimethyl sulfoxide (DMSO) (0 nm TTX) controls all resulted in similar low MTT activities, while the positive control (100 nM TTX) resulted in a clear increase of the MTT activity. These results confirmed that using this specific protocol, the neuroblastoma bioassay could be used for the qualitative screening of large numbers of shellfish samples for the potential presence of voltage gated sodium blocker toxins such as TTXs and PSP toxins in shellfish. These initial findings are promising and therefore the assay is currently being validated for the application towards detecting TTX at regulated levels in shellfish. The presence of relative low levels of PSP toxins (STXs) is expected to cause false positive results as the sensitivity of the bioassay is comparable for STX and TTX and the regulated level of STXs is much higher than that of TTXs, i.e., 800 µg STX 2HCl-eq/kg. However, Dutch shellfish production areas were not affected by PSP toxins over the last 20 years.

**Figure 4:**
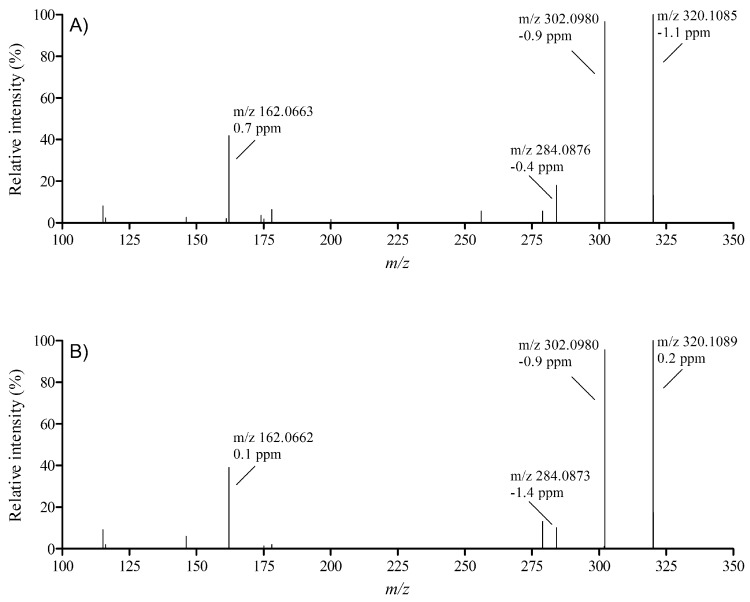
High-resolution MS/MS spectra recorded on a Q-Exactive orbitrap at a resolution of 140,000 full width half maximum (FWHM) of (**A**) standard solution of TTX and (**B**) of TTX in a naturally contaminated oyster sample.

Additionally, a subset of contaminated samples, the same as used for the neuro-blastoma assay, was analyzed by high-resolution mass spectrometry in order to screen for the presence of a broad range of TTXs and other marine biotoxins. The search included 31 different TTX analogues as well as approximately 800 other marine and freshwater toxins. It is well known that for species such as puffer fish and snails, a wide variety of TTX analogues such as deoxy analogues and epimers can be present due to the metabolism in the contaminated organism. The search with high-resolution mass spectrometry did not reveal other analogues than the ones already detected in the targeted LC-MS/MS method. For confirmation, also, high-resolution MS/MS spectra were recorded from both a standard as well as from the contaminated oyster sample (Figure 4). Both precursor *m*/*z* as well as the fragments showed mass deviation errors well below 5 ppm. The contaminated shellfish contained TTX predominantly and only in a single case 4 epi-TTX was detected. The concentration, determined with the LC-MS/MS, of 4 epi-TTX was estimated at 14.5 µg TTX-eq/kg using the TTX calibration curve. While TTX itself was present at 96 µg/kg. The lack of TTX metabolites in shellfish is in line with the findings in Greece and the UK [13,15].

## 3. Discussion 

No action or legislative limit was established for shellfish on both a national or international level at the time of the first positive samples in 2015. After our findings, confirming those of Greece and the UK, discussions on an international level started on safe concentrations for TTX in shellfish. As TTX is a toxin associated with serious intoxications, it was decided in The Netherlands to apply the precautionary principle for the course of 2016. Therefore, the LOQ of the method, which is 20 µg/kg, was used by the competent authority as decision limit for shellfish food safety management to open and close Dutch shellfish production areas. In 2017, the EFSA performed a risk assessment on TTX in shellfish [20]. An acute reference dose (ARfD) for TTX of 0.25 µg/kg body weight was derived, based on effects in mice. This implied that the TTX concentration in a large portion of 400 g shellfish, consumed by a 70 kg person, should not exceed 44 µg TTX/kg shellfish. This safe concentration was adopted in The Netherlands by the risk managers and thus used in 2017 as the decision limit in the shellfish food safety program to open and close Dutch production areas. Looking back at the results since 2015 only 6 samples taken in the sanitary survey program exceeded the limit of 44 µg TTX/kg. Respectively, 3 times in 2015, 2 times in 2016 and only once in 2017. Furthermore, within the sanitary survey samples only oysters exceeded this limit. If these Dutch episodes are placed in perspective with other toxin classes in other countries, the number of closures can be regarded as relatively low and short. However, the closures were problematic, as one of the affected locations was close to the shellfish packaging facilities that are used as a shellfish warehouse. This means that even a short closure has a major financial impact on the shellfish industry.

The toxic episodes seem to be consistent over the last three years with an increase in TTX concentration during late June followed by a rapid decline in July and absence of TTX during the rest of the year. The organism responsible for producing TTX in these areas is not known yet and still under investigation. In other areas such as the UK and Spain, *vibrio* was positively identified as one of the producers of TTX [15,21]. In literature a relationship is suggested between the occurrence of TTX and phytoplankton, which should thus be kept in mind as a possible factor for TTX transport to shellfish too. Other important factors that play a role in TTX production are most likely the climatic and hydrodynamic conditions as suggested by Turner et al. [16]. For example, currents, refreshment rate, water depth, nutrients, bacteria and phytoplankton compositions and numbers, rain fall, water temperature, etc. might be of influence. The relationships of these factors with the TTX concentration in shellfish are unclear and still under investigation. Water temperatures, phytoplankton composition and numbers as well as rainfall conditions were completely different in the three years [22]. Unfortunately, only from 2017 and onwards data are continuously collected on the various climatic and hydrodynamic parameters. Therefore, it will be difficult to retrospectively explain why the maximum TTX concentrations in shellfish in 2015 and 2016 were higher than those observed in 2017. However, when looking at the conditions in the affected areas, there are some factors that seem to be of importance, as the affected areas are relatively shallow with a low refreshment rate of the water. Additionally, the water depth is also low compared to other production areas. Positive relationships between TTX concentrations and low refreshment rates and low depths are in line with findings of Turner et al. [16]. However, more data should be collected in a harmonized way in the coming years to study and identify the parameters involved in TTX production and the organism responsible for producing it.

Regarding toxin analysis, mass spectrometric analysis revealed almost solely TTX and no analogues with the exception of a low concentration of 4-epi-TTX in one sample. This indicates that there is a limited degree of metabolism of TTX in the shellfish itself. It is known that bacterial strains produce mainly TTX however also 4-epi-TTX and anhydro-TTX can be formed [23]. Other metabolites are most likely produced within the liver of for example puffer fish; however TTX and 4,9-anhydro-TTX are often the most predominant metabolites [24]. This simplifies the analytical procedure for mussels, as the TTX certified reference standard also contains low levels of 4-epi and 4,9 anhydro-TTX, while standards for other low abundant metabolites as detected in fish are not available. As already mentioned in the introduction, the use of the MBA in the past might have protected consumers for being exposed to TTX via contaminated shellfish products. The use of alternative methods based on a different principle such as analytical techniques (physiochemical processes) instead of animals (toxicological effect) might overlook these toxins. Therefore, the use of alternative animal-free effect-based bioassays such as the neuro-2a cell viability assay can be useful to detect potential new risks/toxins present in shellfish, as shown in the present study.

## 4. Conclusions

During the course of 2015, 2016 and 2017, TTX was detected in shellfish harvested in Dutch production waters. Oysters seem to be more susceptible than mussels, as the highest concentrations annually found are in oysters, with a maximum concentration of 253 µg TTX/kg shellfish in 2016. Solely TTX was found and no analogues of TTX. In the past the MBA might have protected consumers to high TTX exposure but nowadays TTX can be detected at sufficiently low concentrations by LC-MS/MS. However, when relying solely on analytical techniques, new risks might be missed. To detect known marine biotoxins including TTX and its analogues but also new risks/toxins, the use of effect-based in vitro assays such as the neuro-2a cell viability assay might be of interest. At the Dutch national level, the concentration of 44 µg TTX/kg derived by EFSA was adapted as the action limit. At the European level, no decision is taken yet. From 2016 and onwards TTX is included in the Dutch sanitary survey program and monitored by LC-MS/MS most likely till the neuroblastoma assay is validated and accredited.

## 5. Materials and Methods 

### 5.1. Reagents and Standards

Water was deionized and passed through a Milli-Q water purification system from Merkmillipore, Darmstadt, Germany. Acetonitrile (ULC-MS) and methanol (ULC-MS) were purchased from Biosolve, Valkenswaard, The Netherlands. Ammonium hydroxide (25%), formic acid (98–100%), acetic acid (100%), dimethylsulfoxide (DMSO), *n*-hexane, ouabain and veratridine were purchased from Merck, Darmstadt, Germany. Certified Tetrodotoxin (TTX) material (25.8 ± 2.1 µg/g) was purchased from Cifga, Lugo, Spain. Non-certified TTX material was purchased from Latoxan, Portes lès Valence, France.

### 5.2. Preperation of Extracts

Homogenates of shellfish (mussels, oysters, cockles and ensis) were prepared by homogenizing at least 100 g of whole flesh tissue with a T25 Ultra Turrax mixer at 24,000 rpm (IKA Works, Wilmington, NC, USA). For LC-MS/MS analysis, one gram of homogenate was extracted by vortex mixing during one minute with 1.5 mL 50:50 (*v*/*v*) water/methanol containing 0.015 M acetic acid. Subsequently the extract was centrifuged at 3600 rpm during 5 min. The supernatant was transferred to a volumetric flask of 10 mL and the pellet was extracted for a second time with 2 mL 50:50 (*v*/*v*) water/methanol containing 0.015 M acetic acid. After centrifugation for 5 min at 3600 rpm, the supernatant was transferred to the same volumetric flask already containing previous obtained extract. The volume was adjusted to 10 mL with acetonitrile, and the solution was mixed. After 2 min, this time was allowed for precipitation of proteins, an aliquot of 2 mL was transferred to an ultra-centrifuge filter tube (Amicon Ultra-4 30kD, Merck, Darmstadt, Germany), centrifuged for 30 min at 3,600 rpm. An aliquot of the filtrate was transferred to a LC-MS/MS vial for analysis.

For initial quantitation, a calibration curve was constructed using blank mussel fortified with TTX at respectively 0, 20, 40, 60 and 80 µg TTX/kg shellfish. For a more accurate quantitation, a single concentration standard addition was performed at 60 µg TTX/kg for the samples from the affected areas. If concentrations were much higher, above 100 µg/kg, a second experiment was performed with a higher standard addition concentration.

For the neuroblastoma bioassay, two grams of homogenate was extracted by vortex mixing for one minute with 0.6 mL 2% (*v*/*v*) acetic acid. Afterwards the samples were placed in a 100 °C water bath for 5 min. After cooling to room temperature, the sample was centrifuged at 3600 rpm for 5 min. The supernatant was transferred to a conical calibrated tube and the pellet was extracted a second time with 0.6 mL 2% (*v*/*v*) acetic acid. After vortex mixing, the extract was centrifuged at 3600 rpm and the supernatant was combined with the previous obtained supernatant. The total volume was adjusted to 2 mL with water. To remove matrix effects, a solid phase extraction (SPE) using a Strata-X cartridge (200 mg/6 mL) (Phenomenex, Torrance, CA, USA) was applied. The cartridge was conditioned with 6 mL methanol followed by 6 mL water. Then 1 mL of the crude extract was applied to the cartridge and the eluate was collected in a conical graduated tube. Subsequently, the cartridge was additionally eluted with 2 mL water, the eluate was collected in the same conical graduated tube. Vacuum was applied to retrieve all eluent, thereafter the eluent volume was adjusted to 4 mL with water. Subsequently the obtained eluent was further cleaned using liquid-liquid partitioning by adding 4 mL of hexane. After vigorously mixing the mixture was centrifuged at 3000 rpm for 7 min. The hexane layer was discarded, and 3 mL of the water layer was evaporated in a water bath at 60 °C under a continuous stream of nitrogen. The residue was reconstituted in 300 µL DMSO and filtered using an 0.45 µm mini-membrane filter (Whatman uniprep, Merck, Darmstadt, Germany) prior to the application of the neuroblastoma bioassay.

### 5.3. TTX LC-MS/MS Analysis

The chromatographic method was adapted from Boundy et al. [17]. Briefly, chromatographic separation was achieved using a Waters Acquity I-Class UPLC system (Waters, Milford, MA, USA). The system consisted of a binary solvent manager, sample manager and a column manager. The column temperature was kept set at 60 °C and the temperature of the sample manager was kept at 10 °C. The analytical column used was a Waters BEH Amide (150 × 2.1 mm, 1.7 µm) column, the analytical column was protected by a Waters BEH Amide VanGuard pre-column (5 × 2.1 mm, 1.7 µm). See Boundy et al. for a detailed description of the conditioning and rinsing procedures [17]. Mobile phase A was water containing 0.015% (*v*/*v*) ammonia and 0.015% (*v*/*v*) formic acid and mobile phase B was acetonitrile/water (70:30 *v*/*v*) containing 0.01% (*v*/*v*) formic acid. A gradient started at 2% A at a constant flow rate of 0.4 mL/minute and after 5 min this was linearly increased to 50% A in 2.5 min. This composition was kept for 1.5 min, but the flowrate was linearly increased to 0.5 mL/min. Within 0.5 min the mobile phase composition was returned to 2% A. Subsequently the flow increased to 0.8 mL/minute and was kept for 0.6 min. The flow was linearly decreased to 0.4 mL/minute in 0.4 min prior the next injection. A 2 µL injection volume was used. The effluent was directly interfaced in the electrospray ionization (ESI) source of the Waters TQ-S. The ESI was operated in the positive modus. A capillary voltage of 0.5 kV, a source temperature of 150 °C and a desolvation temperature of 600 °C at a N_2_ flow of 1000 L/h were used. Argon was used as collision induced dissociation gas at a flow rate of 0.25 mL/min. The LC-MS/MS was operated in multiple-reaction-monitoring (MRM) mode, the cone voltage was kept at 40V for all MRM transitions. For TTX (and the 4-epi-TTX) two transitions were monitored *m*/*z* 320.1 > 302.1 (CE 20eV) and *m*/*z* 320.1 > 162.1 (CE 38 eV). Furthermore, the following TTX metabolites were included in the method, 5,6,11 trideoxy TTX *m*/*z* 272.1 > 254.1 (CE 20eV) and *m*/*z* 272.1 > 162.1 (CE 38 eV), 11 nor TTX-6-ol *m*/*z* 290.1 > 272.1 (CE 20eV) and *m*/*z* 290.1 > 162.1 (CE 38 eV), 4,9-anhydro-TTX *m*/*z* 302.1 > 256.1 (CE 20eV) and *m*/*z* 302.1 > 162.1 (CE 38 eV), 5- and 11- deoxy TTX *m*/*z* 304.1 > 286.1 (CE 20eV) and *m*/*z* 304.1 > 176.1 (CE 38 eV). Acceptance criteria for the data were as follows: (1) the constructed calibration curve for TTX should have a correlation above 0.990; (2) sensitivity based on the slope of the calibration curve measured before and after the samples should not deviate more than 25%; (3) the retention times should not deviate more than 0.2 min with the average retention time obtained with the samples used to construct the calibration curves and (4) the calculated ion ratio should not deviate more than 30% with the obtained ion ratios from the samples used to construct the calibration curves. The LOD and LOQ were determined in fortified shellfish extracts. The LOD was determined at a signal to noise of 3 for the transition with the highest intensity (*m*/*z* 320.1 > 302.1) and the LOQ was determined by a signal of noise of 3 for the transition with the lowest intensity (*m*/*z* 320.1>162.1). This will mean that at the LOQ two transitions are available and confirmation based on ion ratio can be performed.

### 5.4. LC-Q-Exactive Orbitrap Screening TTX Analogues

The chromatographic method used is a method developed for the broad screening of marine and freshwater toxins in various matrices such as fish, shellfish, and food supplements. In this case, the method was solely used for screening of TTX and analogues. Chromatographic separation was achieved using a Thermo scientific Ultimate 3000 (San Jose, CA, USA). The column temperature was set at 35 °C and the temperature of the sample manager was kept at 10 °C. The analytical column used was a TSK-Amide-80 (150 × 3 mm, 2 µm) column (Merck, Darmstadt, Germany). Mobile phase A was water and mobile phase B was acetonitrile/water (90:10 *v*/*v*) both containing 0.5 mM formic acid and 2 mM ammonium formate. A gradient started at 10% A at a constant flow rate of 0.5 mL/minute and after 0.1 min this was linearly increased to 55% A in 13.9 min. Then within 0.1 min the composition was changed to 80% A. This composition was kept for 1.9 min. Within 0.1 min the mobile phase composition was returned to 10% A. This composition was kept for 3.9 min prior the next injection. A 10 µL injection volume was used. The effluent was directly interfaced in the heated electrospray ionization (HESI) source of the Thermo Scientific Q-Exactive focus hybrid quadrupole-orbitrap mass spectrometer (San Jose, CA, USA). The ESI was operated in the positive modus. A spray voltage of 3.5 kV, a capillary temperature of 256.25 °C, a probe heather of 412.5 °C and S-Lens of 50 V was set. The data was acquired in full scan (FS) and data independent acquisition (DIA) mode. In FS the scan mass range was set at *m*/*z* 100–1500 with a mass resolution of 70,000 at full width half maximum (FWHM), the automatic gain control (AGC) was set at 1.0e6 and the maximum injection time (IT) 200 ms. For DIA the mass resolution was set at 17,500 at FWHM, AGC at 1.0 × 10^6^ and IT 200 ms and a variable isolation window. The inclusion list contained three DIA windows *m*/*z* 300 with an isolation window of 400 Da, *m*/*z* 750 with an isolation window of 500 Da and *m*/*z* 1250 with an isolation window of 500 Da in every DIA window a normalized collision energy of 40% was applied. Data analysis was performed with Thermo TraceFinder using a library for marine and freshwater toxins, the specific part on tetrodotoxins could be found in the supplementary material (Table S1). To have a suspect hit the precursor ion *m*/*z* should be present with a mass deviation below 5 ppm and at least one fragment ion with a mass deviation of less than 5 ppm should be visible. To test the system a mixture of various hydrophilic toxins is used including TTX for which retention time and mass deviations for the precursor and fragments should full fill the mentioned mass criteria. The mixture also contained a variety of PSP toxins such as saxitoxin, neo-saxitoxin, gonyautoxins,-1,-2,-3,-4 and their decarbomoyl forms but also freshwater toxins such as anatoxins and cylindrospermopsin and also β-methylamino-l-alanine was included. For identification purpose, also, high-resolution MS/MS spectra were recorded from both a standard solution as well as from a high contaminated oyster sample. The *m*/*z* 320.1 was used as precursor mass and spectra were recorded with a resolution of 140,000 FWHM.

### 5.5. Neuroblastoma Assay

Neuroblastoma neuro-2a cells were purchased from the American Type Culture Collection (ATCC; CCL-131) and cultured in 75 cm^2^ culture flasks containing 15 mL RPMI-1640 medium (R0883, Merck, Darmstadt, Germany) supplemented with 10% (*v*/*v*) Fetal Bovine Serum (FBS), 1% (*v*/*v*) of a 100 mM sodium pyruvate solution and 1% (*v*/*v*) of a 200 mM l-glutamine solution. The cell line was maintained in a humidified incubator at 37 °C under 5% CO_2_ and sub-cultured three times per week (dilution 1/14) up to approximately 90% confluence. 

Neuro-2a cells were seeded into 96-well plates with an initial density of 2.5 × 10^4^ cells/well. After growing the cells for 24 h, exposure to a 100 nM TTX standard solution and 20 µL of the previous obtained sample extracts was performed in quadruplicate in 200 μL medium for 24 h. For screening neurotoxic effects, ouabain and veratridine at concentrations that produce an 80% decrease in MTT activity (0.3 mM and 0.03 mM respectively) were added to each well in combination with the test compound or sample extract in fresh serum free medium. The final DMSO concentration in the medium was kept at 0.25% (*v*/*v*) for all standards and extracts. At the end of the exposure, cell viability was measured using the MTT assay. Briefly, 60 μL of MTT (final concentration of 0.8 mg/mL), dissolved in medium were added to each well. After 30 min incubation at 37 °C, the medium was removed, and the formed formazan crystals were dissolved in 100 μL DMSO. The absorbance was measured at 540 nm and corrected for background absorption at 650 nm.

## Supplementary Materials

The following are available online at http://www.mdpi.com/2072-6651/10/11/450/s1, Table S1: TTX HRMS library used.
